# A comprehensive overview of *FCGR3A* gene variability by full-length gene sequencing including the identification of V158F polymorphism

**DOI:** 10.1038/s41598-018-34258-1

**Published:** 2018-10-29

**Authors:** Niken M. Mahaweni, Timo I. Olieslagers, Ivan Olivares Rivas, Stefan J. J. Molenbroeck, Mathijs Groeneweg, Gerard M. J. Bos, Marcel G. J. Tilanus, Christina E. M. Voorter, Lotte Wieten

**Affiliations:** 1 Department of Transplantation Immunology, Tissue Typing Laboratory, GROW School for Oncology and Developmental Biology, Maastricht University Medical Center+, Maastricht, The Netherlands; 2 Department of Internal Medicine, division of Hematology, GROW School for Oncology and Developmental Biology, Maastricht University Medical Center+, Maastricht, The Netherlands

## Abstract

The *FCGR3A* gene encodes for the receptor important for antibody-dependent natural killer cell-mediated cytotoxicity. *FCGR3A* gene polymorphisms could affect the success of monoclonal antibody therapy. Although polymorphisms, such as the FcγRIIIA-V158F and -48L/R/H, have been studied extensively, an overview of other polymorphisms within this gene is lacking. To provide an overview of *FCGR3A* polymorphisms, we analysed the 1000 Genomes project database and found a total of 234 polymorphisms within the *FCGR3A* gene, of which 69%, 16%, and 15% occur in the intron, UTR, and exon regions respectively. Additionally, only 16% of all polymorphisms had a minor allele frequency (MAF) > 0.01. To facilitate (full-length) analysis of *FCGR3A* gene polymorphism, we developed a *FCGR3A* gene-specific amplification and sequencing protocol for Sanger sequencing and MinION (Nanopore Technologies). First, we used the Sanger sequencing protocol to study the presence of the V158F polymorphism in 76 individuals resulting in frequencies of 38% homozygous T/T, 7% homozygous G/G and 55% heterozygous. Next, we performed a pilot with both Sanger sequencing and MinION based sequencing of 14 DNA samples which showed a good concordance between Sanger- and MinION sequencing. Additionally, we detected 13 SNPs listed in the 1000 Genome Project, from which 11 had MAF > 0.01, and 10 SNPs were not listed in 1000 Genome Project. In summary, we demonstrated that *FCGR3A* gene is more polymorphic than previously described. As most novel polymorphisms are located in non-coding regions, their functional relevance needs to be studied in future functional studies.

## Introduction

Natural killer (NK) cells are innate lymphocytes and pivotal players in the defence against malignant- or virally-infected cells^1^. NK cells can produce cytokines and kill target cells^2^. Moreover, NK cells mediate antibody-dependent cell-mediated cytotoxicity (ADCC) via the ligation of their low affinity Fc receptor, FcγRIIIa, also known as CD16a, with an antibody bound to a potential target cell^1,3^.

As reviewed recently, the strength of the ADCC response could be determined by several factors, amongst them the isotype-, fucosylation- and glycosylation- characteristics of the antibody as well as genotypic variation of the FcγRIIIa receptor itself^4^. A clear example of the latter is the single nucleotide substitution (SNP) from G to T at cDNA nucleotide position 559 of the *FCGR3A* gene generating two different FcγRIIIa allotypes: one with a valine (V) and one with a phenylalanine (F) at amino acid position 158, known as FcγRIIIA-V158F polymorphism (rs396991)^5–7^. The presence of a valine (V/V or V/F) has been shown to enhance the NK cell’s binding affinity to an IgG1 or IgG3 antibody as compared to the presence of a homozygous phenylalanine genotype (F/F), resulting in a higher level of NK cell-mediated ADCC^6–8^.

In antibody-based immunotherapy, NK cell-mediated ADCC is one of the mechanisms underlying the anti-cancer effects of frequently used antibodies like rituximab, trastuzumab, and cetuximab. Several clinical studies provided evidence for the functional relevance of the V158F polymorphism in this setting: in non-Hodgkin lymphoma, HER-2/neu-positive metastatic breast cancer, metastatic colorectal cancer or head and neck cancer, patients with V/V polymorphism appeared to have an improved progression-free survival as compared to patients with F/F phenotype^9–13^. Moreover, a study examining rituximab and ADCC in healthy donors suggested that the expression of at least one valine at FcγRIIIa-158 could explain the improved clinical outcome^14^. Nonetheless, two other studies^15,16^ did not find any correlation between the V158F polymorphism and the clinical outcome, possibly due to sample size limitation.

The characterization of the *FCGR3A* gene polymorphism may also be relevant in the solid organ transplantation setting where, in the presence of antibodies against a renal graft, NK cells have been shown to mediate ADCC contributing to graft rejection^17,18^. A recent study on cardiac allograft showed that patients with V/V genotype had an enhanced CD16a expression and were associated with a higher risk of developing vasculopathy and eventually allograft rejection^19^.

Interestingly, a study on bone marrow transplantation for myeloid malignancies suggested that the V158F polymorphism in recipients could predict transplant outcomes and the presence of V/V genotype in recipients was associated with a significantly reduced risk of acute and chronic graft-versus-host disease as well as better overall survival^20^. Furthermore, patients with F/F or V/F genotype have been shown to have a higher predisposition to an increased incidence of infection after liver transplantation^21^.

In addition to the V158F polymorphism, several additional polymorphisms in the *FCGR3A* gene have been identified: (1) the FcγRIIIA-48L/R/H polymorphism (rs10127939), where a single nucleotide substitution from T to G is responsible for a leucine (L) to an arginine (R) substitution and T to A is responsible for a leucine (L) to a histidine (H) at amino acid position 48. Both these substitutions have been reported to have an enhanced binding to the IgG_1_, IgG_3_, and IgG_4_^22^. This polymorphism has also been demonstrated to be linked to the FcγRIIIA-V158F polymorphism^6^ where the FcγRIIIA-48L/R/H polymorphism influenced ligand binding capacity in the presence of the FcγRIIIA-V158F polymorphism^23^. The presence of R or H allele and at least one copy of V allele provided a higher binding capacity. (2) A homozygous missense mutation in the *FCGR3A* gene encoding a L48H substitution causing a defect in NK cell cytotoxicity due to a reduced surface expression of CD2, a co-activation receptor, while preserving an intact ADCC^24^. (3) two SNPs (rs4656317 and rs12071048) located within the enhancer region of the *FCGR3A* gene that are in strong linkage disequilibrium with the FcγRIIIA-V158F polymorphism and strongly affected NK cell ADCC activity where the major alleles had a higher ADCC activity than those with minor alleles^25^, (4) a 3-SNP/1-indel FCGR3A intragenic haplotype which was associated with increased FcγRIIIa expression^26^. (5) Several other polymorphisms in the *FCGR3A* gene, i.e. rs2099684^27^; rs10919543^28^; and rs445509^29^, that have been found to be associated with arteritis^27,28^ and chronic periodontitis^29^.

The above mentioned studies highlighted the potential relevance of *FCGR3A* polymorphisms for NK cell effector function and their potential clinical relevance. However, the analysis is frequently complicated by the presence of *FCGR3B* gene, encoding the inhibitory FcɣRIIIb receptor, as the *FCGR3B* gene is highly homologous to the *FCGR3A* gene except that it has a T at nucleotide 531 of the cDNA instead of a C^7,30^. Another issue is that previous methods were focused on sequencing particular exons of the gene^7,31^ while extended polymorphism in for example 5′ or 3′ UTR or in introns could also influence CD16a expression e.g. by influencing micro-RNA binding or alternative splicing^32^. To facilitate future studies to unravel the functional consequences of CD16a polymorphism, we established a standardized way to determine V158F gene polymorphism using Sanger sequencing and we tested a new full-length gene single molecule sequencing method for the identification of polymorphism in the *FCGR3A* gene using MinION (a Nanopore technology). We subsequently used these methods, combined with the data present for the *FCGR3A* gene in the database of phase 3 of the 1000 Genomes project (1KGP), to generate a more comprehensive overview of full-length CD16a polymorphism.

## Results

### *FCGR3A* gene variability beyond the V158F polymorphism

**Figure 1:**
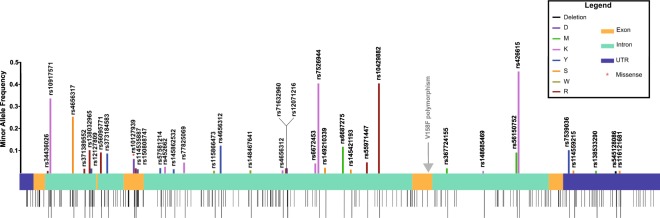
Schematic illustration of *FCGR3A* gene and its 234 polymorphisms according to the 1000 Genome database. The stripes present underneath represent different polymorphisms. All polymorphisms with MAF > 0.01 are shown on the upper part of the scheme and the rs number is shown. Different colors denote the amino acid changes as shown in the legend. The grey arrow points at the location of the V/F polymorphism.

To study the magnitude of *FCGR3A* gene polymorphism, we analysed the nucleotide variability data available in the 1KGP for this gene and mapped all the polymorphisms identified in 1KGP based on the location (gene region) and the minor allele frequency (MAF) (Fig. 1). The polymorphic index (PI), the number of polymorphic positions divided by the length of the whole gene and of the individual introns/exons/UTR, was calculated (Table 1). This illustrated that exon 3 is the most polymorphic region in the gene, with a PI of 0.066, while exon 2 has the lowest PI. The gene sequence of exon 2–5 encodes for the FcγRIIIa receptor, which consists of an extracellular domain with two Ig-like domains (exon 3 and 4) and five potential N-glycosylation sites (three in exon 3 and two in exon 4), a transmembrane domain (exon 5) and a cytoplasmic domain (exon 5).

**Table 1:** Number of polymorphisms present in the *FCGR3A* gene described in the 1KGP database.

Location	Bases	Polymorphisms	PI
5′UTR	183	5	0.027
Exon 1	147	8	0.054
Intron 1	664	30	0.045
Exon 2	20	0	0.000
Intron 2	331	13	0.039
Exon 3	257	17	0.066
Intron 3	3461	87	0.025
Exon 4	257	4	0.016
Intron 4	1501	32	0.021
Exon 5	186	5	0.027
3′UTR	1252	33	0.026
Coding region	867	34	0.039
Noncoding region	7392	200	0.027
Whole gene	8259	234	0.028

The table reports the number of polymorphisms per location and the polymorphic index. PI = Polymorphic index.

**Figure 2:**
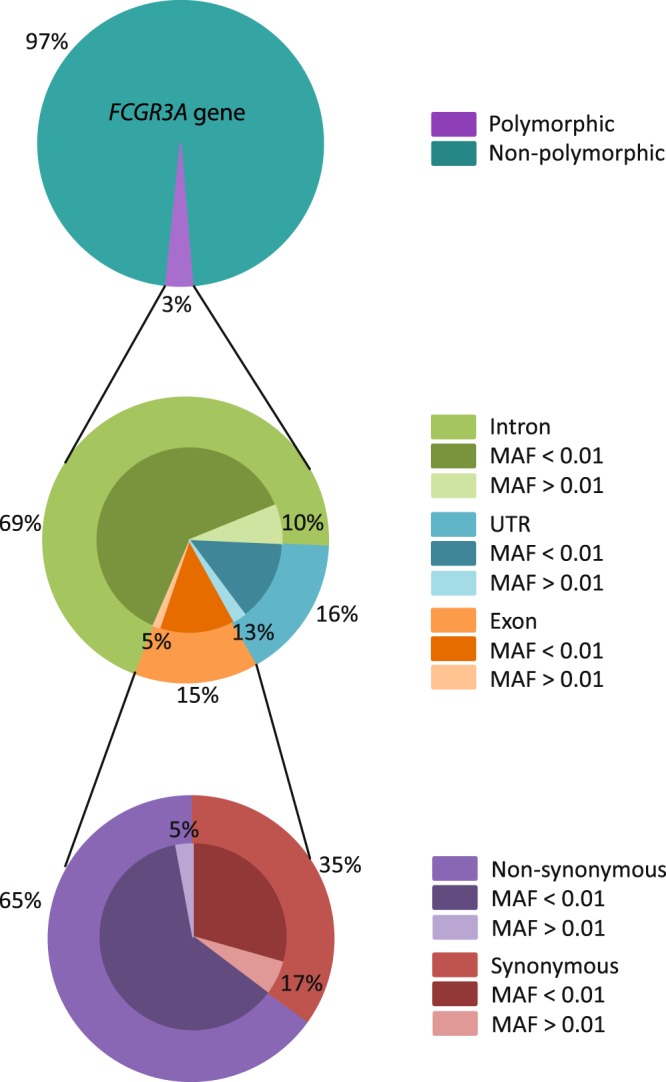
Schematic overview of overall polymorphisms in the *FCGR3A* gene in the 1KGP database. The upper circle depicts the variability percentages of the whole *FCGR3A* gene. The middle circle represents the variability in the specific gene regions and the lower circle shows the exonic variability. The percentages of polymorphisms found with a Minor Allele Frequency (MAF) lower or higher than 1% are specifically illustrated.

A total of 234 polymorphisms (3% of the entire gene, SNP density: 2.83 SNP/100 bp) were identified, of which 34 (15%) are present in the exons, 162 (69%) in the introns, and 38 (16%) in the untranslated regions (UTRs) (Fig. 2). Of note, only 36 of these 234 polymorphisms have a MAF higher than 1% (Table 2). A relatively high number of these polymorphisms with a MAF higher than 1% are located in intron 3 as compared to the other regions (16 out of 36).

**Table 2:** Polymorphisms with a minor allele frequency (MAF) higher than 1% in the 1KGP.

SNP	Position in gene	Location	Polymorphism	MAF	Amino Acid Change
rs34436026	195	Intron 1	R	A: 0.011	
rs10917571	224	Intron 1	K	T: 0.340	
rs4656317	516	Intron 1	S	G: 0.257	
rs371389552	664	Intron 1	R	A: 0.021	
rs138032965	727	Intron 1	R	A: 0.106	
rs12127809	756	Intron 1	Y	C: 0.022	
rs56095771	878	Intron 2	R	G: 0.095	
rs373184583	959	Intron 2	Y	T: 0.090	
rs10127939	1302	Exon 3	D	G: 0.039, A: 0.027	L/R/H
rs114535887	1321	Exon 3	R	A: 0.019	
rs150808747	1336	Exon 3	Y	T: 0.012	
rs57581214	1641	Intron 3	Y	T: 0.024	
rs452662	1696	Intron 3	K	T: 0.030	
rs145862532	1816	Intron 3	Y	T: 0.019	
rs77825069	1950	Intron 3	K	T: 0.048	
rs115866473	2336	Intron 3	W	T: 0.012	
rs4656312	2418	Intron 3	Y	T: 0.126	
rs148467641	2802	Intron 3	W	T: 0.014	
rs145392761	3213	Intron 3	K	T: 0.014	
rs71632960	3275	Intron 3	Y	T: 0.023	
rs12071216	3278	Intron 3	R	G: 0.018	
rs7526944	3678	Intron 3	K	T: 0.408	
rs149210339	3763	Intron 3	S	C: 0.025	
rs6687275	3997	Intron 3	M	C: 0.121	
rs145421193	4098	Intron 3	S	G: 0.017	
rs55971447	4308	Intron 3	R	A: 0.050	
rs10429882	4459	Intron 3	R	G: 0.408	
rs367724155	5333	Intron 4	M	C: 0.023	
rs148685469	5803	Intron 4	K	G: 0.011	
rs56150752	6229	Intron 4	M	C: 0.094	
rs426615	6258	Intron 4	K	G: 0.462	
rs7539036	6904	3′UTR	Y	T: 0.107	
rs114559215	6975	3′UTR	S	C: 0.013	
rs138533290	7256	3′UTR	M	C: 0.012	
rs545128086	7507	3′UTR	Deletion (T/−)	(-): 0.010	
rs116121681	7558	3′UTR	S	C: 0.012	

Of the 34 polymorphisms identified in the exons, 22 (65%) are non-synonymous and 12 (35%) are synonymous. Only one non-synonymous (rs10127939 C/T) and two synonymous polymorphisms (rs114535887 and rs150808747) have a MAF greater than 1%. The non-synonymous polymorphism is located at nucleotide position 1302 in exon 3 with three different nucleotides possible (T, G, and A), resulting in three different amino acids: leucine (L, MAF 0.09), arginine (R, MAF 0.039), and histidine (H, MAF 0.027) and three different alleles. The synonymous polymorphisms are located at nucleotide position 1321 and 1336 and have a MAF of 0.019, and 0.012 respectively.

The V158F polymorphism at nucleotide position 5093 (rs396991) is not documented in the 1KGP because it did not reach the quality control threshold, most probably because of its location in a homopolymer-rich region, and thus no frequency information of this polymorphism was attainable. For this reason, Fig. 1 shows an arrow demonstrating the location of the V/F polymorphism, but provides no further information.

### Sanger sequencing for detection of V158F polymorphism in the *FCGR3A* gene

**Figure 3:**
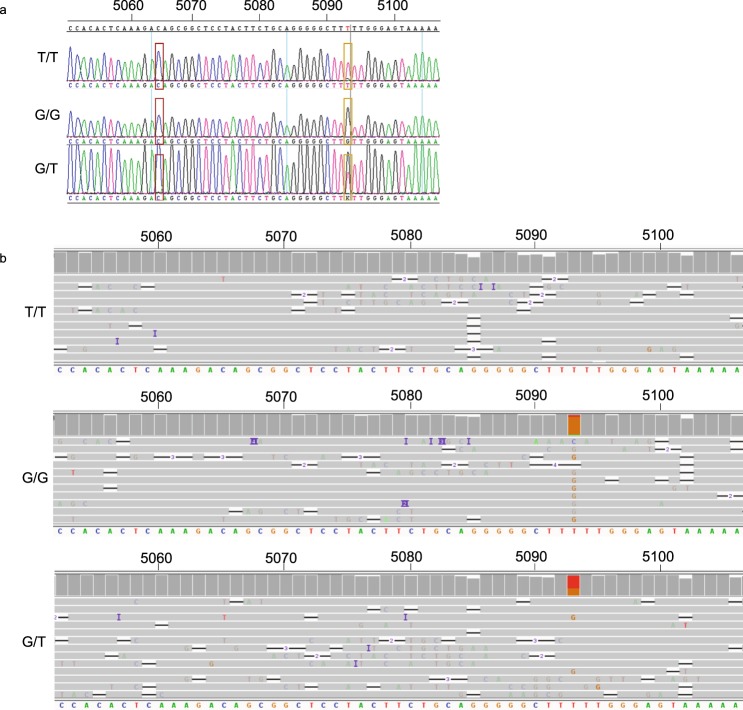
Detection of V158F polymorphism by showing 3 different genotypes, homozygous T, homozygous G, and heterozygous. (**a**) Electropherograms show the Sanger-based sequencing result around the V158F polymorphism. The red square indicates nucleotide position 5064, which is used to check whether the *FCGR3B* gene is co-amplified. The yellow square indicates nucleotide position 5093, used for determining the V158F polymorphism. Nucleotide code K indicates that both T and G are present. (**b**) MinION sequencing result around the V158F polymorphism. Dark grey bars on the top show the sequence coverage identical to the consensus sequence. If the sequence is not identical to the consensus the bars will have the color of the corresponding nucleotide. The light grey lines show a small part of the reads obtained with the MinION run and the sequence at the bottom shows the consensus sequence. The first result represents a sample homozygous for T at position nucleotide 5093 (coverage: A: 0%: C: 2% G: 2% T: 96%), which was also used as consensus, the second sample is homozygous for G (coverage: A: 3% C: 2% G: 85% T: 9%), and the third is heterozygous at nucleotide position 5093 (coverage: A: 1% C: 3% G: 32% T: 64%).

To establish a convenient method to analyse FcγRIIIa-158 polymorphism, while excluding the highly homologous *FCGR3B* gene, we explored a Sanger sequencing based approach that would also enable analysis of extended polymorphism. The *FCGR3A* gene sequence of the 1KGP database was used as a reference for the sequencing analysis. First, we focused the analysis on the FcγRIIIa-158 polymorphism and, in our sequence data, we identified the *FCGR3A* gene by the presence of a C nucleotide at the position 5065 while a T would be identified in case of an *FCGR3B* gene (Fig. 3a). The FcγRIIIa-158 polymorphism variants at nucleotide position 5093 (T/T, T/G, and G/G) could be distinguished by analysis of the chromatograms. The T/T genotype would result in an F/F phenotype (low affinity), T/G in a V/F phenotype, whereas G/G would result in a V/V phenotype (high affinity). We subsequently analysed a total of 76 samples for the V/F polymorphism and a total of 29 samples were found to be homozygous for T (F/F), 42 samples were heterozygous (V/F), and 5 samples were homozygous for G (V/V). Hence, the T allele was overall the most prevalent; 66% T compared to 34% G. To validate the results obtained from the sequencing, we used sequence-specific primers (SSPs) to specifically amplify the variants of the *FCGR3A* gene separately. For this validation, we selected a total of 11 samples. The gel electrophoresis results confirmed the sequencing results (Supplementary Fig. S1).

Altogether, these data showed that the developed Sanger sequencing approach is reliable to identify polymorphisms in the *FCGR3A* gene.

### Detection of extended, full-length polymorphisms is feasible using Sanger- and MinION Nanopore-based sequencing

The results from the 1KGP database analysis on *FCGR3A* gene polymorphisms revealed that there were more polymorphisms within the *FCGR3A* gene than previously described. Additionally, the 1KGP polymorphism frequency database showed that some of these polymorphisms occurred in the worldwide population with a frequency higher than 1.0%. We therefore envisioned that detection of extended full-length polymorphisms, including the non-coding regions, in this gene could facilitate future studies on the functional relevance of the FcγRIIIa receptor polymorphism. To investigate the feasibility of detecting polymorphisms in the *FCGR3A* gene, we set up a pilot study and amplified the whole *FCGR3A* gene region for 14 DNA samples and subsequently sequenced using two approaches: Sanger- and MinION sequencing (Oxford Nanopore Technologies). Despite full-length amplification, we did not perform full length sequencing for Sanger sequencing for this pilot and used primers covering a part of intron 3, exon 4, intron 4, and 3′UTR region. MinION is a novel portable real-time single molecule sequencing device developed to sequence long regions with ultra-long reads. With this technique, we were therefore able to sequence the complete full-length gene, also including all non-coding gene regions. MinION amplification primers were also tagged enabling us to barcode and sequence multiple samples simultaneously. After sequencing, we analysed the sequencing results and compared the results obtained by Sanger sequencing with those obtained by MinION and with the data from the 1KGP.

In this pilot study, we detected 23 SNPs in the *FCGR3A* gene of the 14 individuals (Table 3). Of these 23 SNPs, 13 were also identified by the 1KGP and two of these SNPs (G3121A and T3155C) were found with a MAF < 0.01. The only exonic SNP T5093G (V158F polymorphism) is listed in 1KGP database as “failed variant” and the allelic frequency data is not available in this database. We therefore used the MAF data from other databases (GO-ESP and ExAC) in Table 3. The 10 SNPs not identified by the 1KGP were located in the non-coding intron 3, intron 4 or 3′UTR region.

**Table 3:** SNPs found within the *FCGR3A* gene detected by Sanger sequencing and MinION, compared to 1KGP.

SNP	Chromosomal position	Gene position	Gene location	Detected by			SNP name	MAF
				Sanger	MinION	1KGP		
G224T	1:161519411	224	Intron 1	No*	Yes	Yes	rs10917571	0,34
C516G	1:161519119	516	Intron 1	No*	Yes	Yes	rs4656317	0,26
G727A	1:161518908	727	Intron 1	No*	Yes	Yes	rs138032965	0,11
G1463A	1:161518172	1463	Intron 3	No*	Yes	No	n/a	n/a
G1793C	1:161517842	1793	Intron 3	No*	Yes	No	n/a	n/a
C2418T	1:161517217	2418	Intron 3	No*	Yes	Yes	rs4656312	0,13
A2967G	1:161516668	2967	Intron 3	No*	Yes	No	n/a	n/a
G3121A	1:161516514	3121	Intron 3	No*	Yes	Yes	rs545876704	<0,01
T3155C	1:161516480	3155	Intron 3	No*	Yes	Yes	rs180923798	<0,01
A3187G	1:161516448	3187	Intron 3	No*	Yes	No	n/a	n/a
G3624T	1:161516011	3624	Intron 3	Yes	Yes	Yes	rs6672453	0,11
G3683T	1:161515952	3683	Intron 3	Yes	Yes	Yes	rs7526944	0,41
G3763C	1:161515872	3763	Intron 3	Yes	Yes	Yes	rs149210339	0,03
A4083G	1:161515552	4083	Intron 3	Yes	Yes	No	n/a	n/a
A4327C	1:161515308	4327	Intron 3	Yes	Yes	No	n/a	n/a
A4459G	1:161515176	4459	Intron 3	Yes	Yes	Yes	rs10429882	0,41
T5093G	1:161514542	5093	Exon 4	Yes	Yes	Yes	rs396991	0.27**–0.33***
T5728C	1:161513907	5728	Intron 4	Yes	Yes	No	n/a	n/a
C5876G	1:161513759	5876	Intron 4	No*	Yes	No	n/a	n/a
T6187C	1:161513448	6187	Intron 4	No*	Yes	No	n/a	n/a
T6258G	1:161513377	6258	Intron 4	No*	Yes	Yes	rs426615	0.46
C6904T	1:161512731	6904	3′UTR	Yes	Yes	Yes	rs7539036	0.11
G8054C	1:161511581	8054	3′UTR	No*	Yes	No	n/a	n/a

Using the same amplification primers, 14 DNA samples were sequenced using Sanger and MinION. MAF represents the Minor Allele Frequency data from 2504 individuals obtained from the 1KGP database except for rs396991 (F158V) were the MAF was obtained from the GO-ESP** and ExAC*** database. No* = this position was not included within the Sanger sequence region.

Of the 23 detected SNPs, nine were identified by both the Sanger sequencing and MinION technique. Since the other 14 SNPs were located in regions outside the Sanger sequence area, these were only identified by MinION. This result demonstrates that MinION sequencing can be used to determine full-length *FCGR3A* polymorphism. Although the results of MinION sequencing were similar to Sanger sequencing, some cautions should be taken when reading the sequencing results in the region where V158F polymorphism is located (Fig. 3b). We observed that MinION could misreport the presence of heterozygous G/T where it would be reported as a homozygous T/T genotype (depending on the analysis settings/percentage of nucleotides present), most likely due to the presence of a homopolymer sequence within the region. This might actually be the reason why it is reported as a “failed variant” in the 1KGP, since all NGS methods encounter difficulties in analysing homopolymer regions.

Altogether, we demonstrated that using full-length Sanger-based and MinION-based sequencing methods we could detect both known as well as new polymorphisms within the *FCGR3A* gene.

## Discussion

NK cells are the principal mediator of ADCC due to the high expression of the activating FcγRIIIa and the absence of the inhibitory FcγRIIIb on their surface^3^. The large availability of clinical grade antibodies triggering ADCC against cancer cells has put increased focus on NK cell-mediated ADCC and emphasizes the relevance of the FcγRIIIa for cancer immunotherapy^4^. In addition, a few studies underlined the functional relevance of FcγRIIIa in the transplantation setting by showing that NK cell mediated ADCC could play a role in allograft rejection^33^. Albeit several *FCGR3A* gene polymorphisms have been shown to impact NK cell mediated ADCC, full-length gene polymorphism has not been determined. Hence, we provide here an overview of *FCGR3A* gene polymorphisms, as well as two improved sequencing methods for further gene exploration.

In this study, with 234 polymorphisms identified, we demonstrated that *FCGR3A* gene is more polymorphic than currently known; 34 SNPs were located in the exons and only 3 of them had a MAF > 0.01. We identified two non-synonymous SNPs either by Sanger sequencing/MinION sequencing or in 1KGP. The first one was rs10127939, representing the FcγRIIIA-48L/R/H previously shown to influence ADCC^22,23^. We did not detect this polymorphism in our full-length sequencing samples presumably because of our limited sample size and the fact that the frequency of this polymorphism is relatively low in the population (MAF = 0.039 and 0.027). The second non-synonymous SNP in the coding region was rs396991, representing the V158F polymorphism which we detected both by Sanger- and by MinION sequencing. In our test panel the V/F phenotype (G/T genotype) is the most common (55%) followed by F/F (T/T genotype, 38%) and V/V (G/G genotype, 7%). The presence of V158F polymorphism has been previously investigated in individuals from different populations, including ethnic groups from Singapore^34^, the Netherlands, Great Britain, Norway^35^ and Japan^36^. Overall these studies reported the V/F or F/F phenotype as the most frequent, whereas the V/V was the least frequent phenotype in all populations, which is comparable to our results and could suggest some kind of selective pressure on *FCGR3A*. Our study set up did not allow us to reliably compare V158F gene- and allele frequencies between the 34 samples from the Guadaloupe population vs the 42 samples from our institute or with the results from the 1KGP. The major reason for this was the lack of information on the exact ethnic background of the individuals and the low sample size. Given the known highly heterogeneous background of the Guadaloupe population, it would, however, be highly interesting to compare this population with other populations in a future study.

In this study, we demonstrated that the majority of *FCGR3A* gene polymorphism is located in the non-coding regions and at least 33 of the 200 identified non-coding SNPs have a MAF > 0.01 in the 2504 individuals of the 1KGP. As introns have been demonstrated to be involved in gene regulation^37^ and many intronic polymorphisms could exhibit functional significance^38^, it might be worthwhile to perform additional functional studies. SNPs located in the intron regions could potentially affect RNA splicing by altering the sequences of the 5′/3′ splice site, branch point, polypyrimidine tract or intronic splicing enhancer/silencer motifs. A study on *FCGR2C* gene interestingly showed that a mutation in an intronic splice site introduced novel stop codons resulting in a loss of FcγRIIc expression^39^. In this study we have investigated the two consensus splice site sequences on the 5′ and 3′ end of the intron (GT on 5′ and AG on 3′) and we already observed one SNP (rs544630563) at the 3′ of intron 4, turning AG into GG, although it was found with low frequency in the 1KGP database (MAF < 0.01). Additionally, as in a recent paper reviewing different studies on different disease genes, several mutations deep within the introns (for example 100 base pairs upstream exon-intron boundary) were identified as being associated to multiple diseases^40^. In line with our data, where we observed intronic polymorphisms located upstream the exon-intron boundary, it would be interesting to look at the association of these polymorphisms with the functionality of the FcγRIIIa receptor.

In the present study, we successfully set up a Sanger- and a MinION-based protocol to sequence the *FCGR3A* gene. We subsequently demonstrated that both Sanger sequencing and MinION were able to identify *FCGR3A* polymorphisms present in the 1KGP database. While Sanger sequencing is based on the capillary electrophoresis, MinION technology consists of nanopores embedded in an electrically resistant membrane through which a current is applied, causing a potential which flows through the aperture of the nanopores. The changes observed in the current correspond with 5 to 6 nucleotides passing through the nanopores. This electrical signal is translated into reads that can be analysed and by this technology, MinION can sequence reads up to hundreds of kilo base pairs. For both techniques, we performed identical full-length amplification of the gene. However, MinION has the advantage of directly generating full-length gene reads and phasing of the two variants is possible without group-specific amplification. Although MinION allows full-gene sequencing of various samples in a relatively short time, this technology is not yet widely implemented. Compared to conventional sequencing approaches MinION has a lower accuracy and sensitivity and therefore more reads must be generated. We demonstrated a good concordance between Sanger sequencing and MinION, and were able to identify the V158F polymorphism in all samples using both MinION and Sanger. Nonetheless, a homopolymer region, such as the sequence around V158F, is known to be problematic in all next generation sequencing approaches, apparently including MinION and presumably this also explains the lack of data for this region in the 1KGP. The challenge of analyzing such homopolymer regions with MinION sequencing has been described in several other studies as well^41–43^. Hence despite its usefulness for full-length gene analysis, Sanger sequencing for now seems the preferred method when only analysis of V158F polymorphism is required.

In summary, we showed that *FCGR3A* gene is highly polymorphic especially in the non-coding regions of the gene requiring functional studies to investigate the functional consequences. Additionally, we demonstrated that our Sanger- and MinION-based sequencing approaches can be used to identify the extended polymorphisms of the gene. Although further optimization and validation is warranted, we also identified MinION as a powerful method to perform direct full-length *FCGR3A* gene sequencing.

## Material and Methods

### Subjects

*FCGR3A* sequences were studied in a test panel consisting of 76 distinct samples with unknown DNA sequence, 42 of them were volunteers from the institute and 36 were individuals from the Guadeloupe islands^44^. Samples were left over from diagnostic procedures which does not require ethical approval in the Netherlands under the Dutch Code for Proper Secondary Use of Human.

### DNA isolation

Genomic DNA was extracted from ethylenediamine tetraacetic acid (EDTA) blood samples using the QIAamp DNA blood mini kit (Qiagen, Hilden, Germany). DNA concentrations were measured using the NanoDrop ND-1000 spectrophotometer (Thermo Scientific, Wilmington, Delaware).

### Amplification of the *FCGR3A* gene for Sanger- and MinION sequencing

#### Amplification primers

Primers specific for the *FCGR3A* gene were designed by comparing the sequences of the *FCGR3A* and *FCGR3B* genes, including their polymorphisms, and finding the discrepancies among them. Due to the extreme homology of the genes, some generic primers (not specific for the *FCGR3A* gene) were also designed as a control and always used in combination with a specific primer.

#### Polymerase Chain Reaction

The entire *FCGR3A* gene, including the 5′UTR and 3′UTR, was amplified using an *FCGR3A* gene-specific forward primer and a generic reverse primer, producing a 9654 bases long polymerase chain reaction (PCR) product. The PCR reaction contained 300 ng of genomic DNA, 67 mM Tris-HCl (pH 8.8) (Merck, Darmstadt, Germany), 16.6 mM ammonium sulfate (Merck), 0.01% Tween 20 (Merck), 1.5 mM MgCl_2_ (Life Technologies, Austin, Texas), 0.2 mM of each dNTP (GE Healthcare, Diegem, Belgium), 0.1 µg/µl cresol red (Sigma-Aldrich, St. Louis, Missouri), 5% glycerol (Alfa Aesar, Karlsruhe, Germany), 15 pmol of each primer (Sigma-Aldrich) and 2.5 U of Expand Long Template PCR System (Roche, Basel, Switzerland) with a final volume of 30 µl. The PCR program consisted of an initial denaturation step of 2 minutes at 94 °C; followed by 10 cycles of 15 seconds at 94 °C, 30 seconds at 63 °C and 4 minutes at 68 °C; then 10 cycles of 15 seconds at 94 °C, 30 seconds at 60 °C and 6 minutes at 68 °C; afterwards 10 cycles of 15 seconds at 94 °C, 30 seconds at 60 °C and 10 minutes at 68 °C; and a final elongation step of 7 minutes at 68 °C. The PCR products were checked by electrophoresis using a 1.5% agarose gel containing 0.5 µg/µl ethidium bromide (Sigma-Aldrich).

#### MinION amplification

The same amplification primers used for Sanger sequencing were used for the MinION sequencing mixture, with a tag-sequence (indicated as italic and red) added to the ends to enable barcoding amplification for identification of different samples after all samples were pooled.

### Sanger sequencing of V158F region

Amplicons obtained from all 76 DNA samples were purified by ExoSAP-IT (Affymetrix, Santa Clara, California) following the manufacturer’s protocol.

Purified amplicons were sequenced using ABI BigDye Terminator Chemistry (Life Technologies) and an ABI 3730 sequencer (Life Technologies) with a forward and a reverse sequencing primer. For *FCGR3A* gene sequencing using Sanger, several sequencing primers were used to cover different locations in the gene. The sequencing mixture consisted of 1 µl purified PCR product, 0.5 µl sequencing primer (5 pmol, Sigma-Aldrich), 1 µl of BigDye Terminator v1.1 mix, 1.5 µl 5x Big Dye Terminator sequencing buffer and 6 µl distilled water. The PCR program consisted of: 1 minute at 95 °C, followed by 25 cycles of 10 seconds at 95 °C, 5 seconds at 50 °C, and 4 minutes at 60 °C. Successively, the mixtures were purified by Sephadex G-50 Fine (GE Healthcare Life Sciences, Little Chalfont, UK) and placed in the ABI 3730 sequencer for capillary electrophoresis sequencing. The chromatograms were aligned with a reference sequence obtained from the 1KGP and analysed using DNASTAR Lasergene SeqMan Pro (DNASTAR Lasergene, Madison, Wisconsin).

### MinION Nanopore-based sequencing

Amplicons obtained from 14 DNA samples from the cohort of individuals present in the institute, were barcoded and sequenced following Oxford Nanopore’s instructions (NSK-LSK208). In short, we purified the amplicons using CleanPCR beads (GC Biotech, Alphen aan den Rijn, the Netherlands) followed by determining DNA concentration using a DS-11 spectrophotometer (DeNovix, Delaware, USA). Next, 48 ng of amplicon was barcoded using the PCR barcoding Kit 1 (Oxford Nanopore Technologies, Oxford, UK) and LongAmp Taq2x (New England Biolabs, Massachusetts, USA) followed by purification of the barcoded PCR product using CleanPCR beads and determination of DNA concentration. The barcoded DNA samples were pooled to an end volume of 1 µg in 45 µl and an endrepair/dA-tailing was performed (NEBNext Ultra II End-Repair/dA-tailing module, New England Biolabs) followed by a purification step using AMPure XP beads (Beckman Coulter, California, USA). After that, DNA adapter ligation was performed using NEB Blunt/TA ligase master mix (New England Biolabs) and samples were purified using MyOne C1 Dynabeads (Thermo Fisher Scientific, Massachusetts, USA). Of this adapter library, 75 µl was loaded into a FLO-MIN106 flow cell. Sequencing run was performed and base calling was done using Albacore software (V1.2.4, Oxford Nanopore Technologies). Sequencing data was analysed using in-house software and Integrative Genomics Viewer (IGV)^45^.

### PCR amplification using sequence specific primers for Sanger sequencing validation

The sequence specific primers (SSPs) consisted of one primer specific for the T allele and one for the G allele. An *FCGR3A* gene-specific primer was used in combination with the SSPs to assure specific amplification of the *FCGR3A* gene. The PCR program was almost identical to the Sanger sequencing amplification protocol described in this article, except that the annealing temperature used for SSP PCR was 63 °C during the first 10 cycles.

### 1000 Genome Project Data Analysis

Based on the publicly available data present in the third phase of the 1KGP (http://phase3browser.1000genomes.org/index.html), including 2504 individuals originating from 26 different populations, the *FCGR3A* gene comprised 8259 bp located on chromosome 1: 161511549-161519818 (reverse direction). This sequence corresponds to the FCGR3A-001 protein coding transcript and the start of exon 1 (position 1:161519634) was used as nucleotide position 1 in this paper. We recorded the polymorphism and its location on the chromosome as well as the gene location (position of nucleotide and region (i.e. UTR, exon, or intron)). The population genetic tool was used to acquire an overview of the overall allele frequencies and the frequencies within different population.

**List of primers**

**Table:** 

Sanger sequencing Amplification Primers		
Direction	Sequence (5′ to 3′)	Location 1000 Genomes
FW	GCTGCCTGGGTTCATTTCCA	1:161520918-161520938
RV	CCTCTGCCCAGGCCTCTA	1:161511283-161511301

MinION Amplification Primers		
Direction	Sequence (5′ to 3′)	Location 1000 Genomes
FW	*TTTCTGTTGGTGCTGATATTGC*GCTGCCTGGGTTCATTTCCA	1:161520918-161520938
RV	*ACTTGCCTGTCGCTCTATCTTC*CCTCTGCCCAGGCCTCTA	1:161511283-161511301

Sanger sequencing V158F region Sequencing Primers		
Direction	Sequence (5′ to 3′)	Location 1000 Genomes
FW	GTGTTCAAGGAGGAAGACC	1:161514701-1:161514719
RV	ACTCAACTTCCCAGTGTGATT	1:161511283-161511301

SSP Amplification Primers			
Direction	Sequence (5′ to 3′)	Location 1000 Genomes	Specificity
RV	AAGACACATTTTTACTCCCAAA	1:161514521-1:161514542	T allele
RV	AAGACACATTTTTACTCCCAAC	1:161514521-1:161514542	G allele
FW	GCTGCCTGGGTTCATTTCCA	1:161520918-161520938	*FCGR3A*

Sanger sequencing *FCGR3A* gene Sequencing Primers			
Direction	Sequence (5′ to 3′)	Location 1000 Genomes	Specificity
FW	CTAATAATGATTCATCTCTYTGC	1:161525783 - 1:161525805	Intron 3
FW	TGCTKAAAAAGTAAGTGGWTAG	1:161525803 - 1:161525824	Intron 3
RV	GGTAAGTATTATAATGGCAYAAG	1:161526243 - 1:161526260	Intron 3
RV	TTATAGGTAAGTATTATAATGGC	1:161526248 - 1:161526265	Intron 3
FW	KTTTGGCAGTGYCAACCWTC	1:161528867 - 1:161528886	Exon 5/3′ UTR
FW	TCCACCTGGGTACCAAGTC	1:161528898 - 1:161528916	Exon 5/3′ UTR
RV	TTCTATGTTTCCTGCTGCTTG	1:161529146 - 1:161529166	Exon 5/3′ UTR
RV	RGGATCTGGCTCTGAGTTC	1:161529163 - 1:161529182	Exon 5/3′ UTR
FW	GTGTTCAAGGAGGAAGACC	1:161514701-1:161514719	V158F region
RV	ACTCAACTTCCCAGTGTGATT	1:161514701-1:161514719	V158F region

## Electronic supplementary material

Supplementary Information

## Data Availability

All data generated or analysed during this study are included in this published article (and its Supplementary Information file).
