# Supplementary material for: A comprehensive overview of FCGR3A gene variability by full-length gene sequencing including the identification of V158F polymorphism
Source: Sci Rep. 2018 Oct 29;8:15983. doi: 10.1038/s41598-018-34258-1 (PMC6206037; doi:10.1038/s41598-018-34258-1)
Supplement: Supplementary file 1 — Supplementary Information [file 41598_2018_34258_MOESM1_ESM.pdf]

Supplementary Information

A comprehensive overview of *FCGR3A* gene variability by full-length gene sequencing including the identification of V158F polymorphism

N.M. Mahaweni[1,2], T.I. Olieslagers[1], I. Olivares Rivas[1], S. J. J. Molenbroeck[1], M. Groeneweg[1], G. M. J. Bos[2], M. G. J. Tilanus[1], C. E. M. Voorter[1], L. Wieten[1]

[1] Department of Transplantation Immunology, Tissue Typing Laboratory, GROW School for Oncology and Developmental Biology, Maastricht University Medical Center+, the Netherlands
[2] Department of Internal Medicine, division of Hematology, GROW School for Oncology and Developmental Biology, Maastricht University Medical Center+, the Netherlands

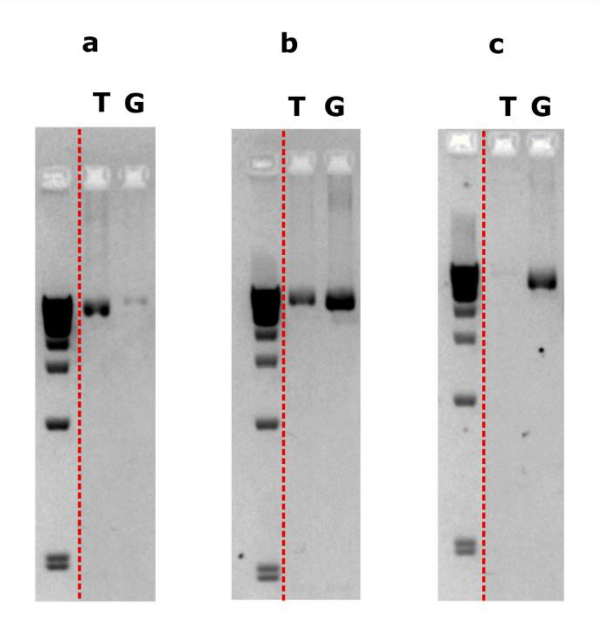

Supplementary Figure S1. The detection of V/F polymorphism by SSP showing 3 different genotypes.

Three different DNA samples representing homozygous T (a), heterozygous (b), and homozygous G (c) genotype. T on the lane means the SSP used for amplification was specific for the T allele, while a G indicates the SSP used for amplification was specific for the G allele. Each figure was derived from 3 different gels. Red dotted lines marked the border between the ladder and the samples.
